# Global trends in biomarker research for periprosthetic joint infection: a bibliometric analysis

**DOI:** 10.1186/s42836-025-00359-2

**Published:** 2026-01-27

**Authors:** Bo Li, Xing Yun, Liang Liu, Zulipikaer Maimaiti

**Affiliations:** Department of Orthopedics, Beijing Luhe Hospital, Capital Medical University, Beijing, 101149 China

**Keywords:** Periprosthetic joint infection, Biomarkers, Bibliometric analysis, α-defensin, Calprotectin

## Abstract

**Background:**

Periprosthetic joint infection (PJI) remains a major diagnostic challenge, and no single biomarker provides definitive accuracy. With rapid advances in synovial, serum, and molecular assays, a comprehensive overview of global biomarker research is needed. This study provides a broad, data-driven mapping of PJI biomarker research, clarifying major thematic shifts and their implications for clinical translation.

**Methods:**

A literature search of the Web of Science Core Collection (2011–2024) identified research and review articles on PJI diagnostics and biomarkers. Bibliometric indicators, collaboration networks, and keyword co-occurrence were analyzed using VOSviewer, CiteSpace, and Bibliometrix. Co-citation and keyword analyses were used to determine influential references and evolving hotspots. Recent high-impact studies and consensus guidelines were reviewed to contextualize the findings.

**Results:**

PJI biomarker publications increased markedly, rising from fewer than five per year before 2014 to 57 in 2020. The 380 papers included accumulated more than 5,200 citations (mean 13.8 per article). China (103) and the USA (88) accounted for half of all output, with the USA showing the strongest citation impact; Germany, the UK, and Italy were also key contributors. Collaboration mapping highlighted Parvizi, Trampuz, and the Rothman Institute as central nodes. The *Journal of Arthroplasty* published the largest share of studies, while *JBJS-Am* and *CORR* had the highest citations per article. Keyword evolution showed a transition from conventional serum markers (2011–2015) to synovial α-defensin and leukocyte esterase assays (2016–2018), and more recently to synovial calprotectin, machine learning, microfluidics, and molecular diagnostics (2019–2024).

**Conclusion:**

From 2011 to 2024, PJI biomarker research grew rapidly, driven mainly by institutions in the United States, China, and Europe. Key themes included synovial α-defensin, calprotectin, machine learning, and next-generation sequencing. Future progress depends on multicenter validation, assay standardization, and integrating biomarkers into diagnostic algorithms. Stronger collaboration, data sharing, and decision-support tools will be essential for earlier and more accurate PJI diagnosis.

Video Abstract

**Supplementary Information:**

The online version contains supplementary material available at 10.1186/s42836-025-00359-2.

## Introduction

Periprosthetic joint infection (PJI) remains one of the most serious complications following total joint arthroplasty (TJA), adversely affecting patient health and imposing considerable economic burdens on healthcare systems [1]. Although the incidence of PJI is relatively low, intensive preventive efforts in recent years have not significantly reduced its occurrence [2]. Among patients undergoing total hip (THA) or total knee arthroplasty (TKA), reported rates vary across studies but generally range from 1 to 2% [3, 4]. As the global population ages and demand for joint replacement continues to grow, the absolute number of PJI cases is expected to rise [5]. Beyond its epidemiological and economic impact, PJI is notoriously difficult to diagnose at an early stage. Limited sensitivity and specificity of conventional microbiological culture techniques, the increasing prevalence of multidrug-resistant organisms, and the nonspecific or subclinical presentation of some cases frequently delay timely diagnosis and treatment, increasing the risk of reoperation and compromising both functional outcomes and patient prognosis.

Recent advancements in biomarker research for PJI reflect a paradigm shift toward precision diagnostics. Currently, several inflammatory and immunological biomarkers have been investigated for the detection and monitoring of PJI, from the traditional serum markers like C-reactive protein (CRP) and erythrocyte sedimentation rate (ESR) to a range of synovial fluid analytes such as calprotectin, lactoferrin, myeloid nuclear differentiation antigen (MNDA), neutrophil gelatinase-associated lipocalin (NGAL), and novel markers like urinary peptide markers and lipocalin-2 [6–8]. Cytokine panels, such as those including IL-6 and GM-CSF, have also shown enhanced performance [9], and there is growing interest in combining multiple biomarkers within integrated algorithms to further improve accuracy [10, 11].

However, no single biomarker has achieved universal acceptance as a “gold standard” for PJI diagnosis. Reported diagnostic thresholds vary considerably across studies and commercial assays, performance may decline in low-grade or culture-negative infections, and some of the most promising tests remain costly or not widely accessible in routine practice. The heterogeneity of patient profiles, infecting organisms, and clinical settings further contributes to variable biomarker performance. This uncertain landscape was underscored in the updated PJI consensus standards from the 2018 International Consensus Meeting (ICM) and the 2021 European Bone and Joint Infection Society (EBJIS), which incorporate biomarkers into multiparametric criteria but do not identify a single dominant test, thereby driving ongoing exploration of mechanism-based biomarkers and novel diagnostic strategies.

Bibliometrics, a methodology that combines quantitative metrics and visualization techniques to map and evaluate scholarly impact, has become an indispensable tool in medicine and life sciences [12]. Unlike narrative reviews that qualitatively synthesize selected studies, bibliometric analyses of PJI biomarker research systematically quantify global research output, collaboration patterns, institutional and author-level impact, and the temporal evolution of research themes. These analyses are essential for shaping evidence-based policies, optimizing resource allocation, and addressing existing research gaps while fostering interdisciplinary and cross-institutional innovation. To our knowledge, no bibliometric study has comprehensively evaluated trends in PJI biomarker research. By addressing this gap, we aim to provide a data-driven mapping of this research domain and to propose future directions for developing and clinically applying biomarkers to facilitate earlier diagnosis, improve treatment outcomes, and ultimately advance the management of PJI.

## Materials and methods

### Data sources and search strategies

This bibliometric study utilized the Web of Science Core Collection (WoSCC) as the primary data source, given its comprehensive coverage of peer-reviewed journals and consistent indexing standards. Publications from January 1, 2011, to December 31, 2024, were included, with the search conducted on January 15, 2025, to ensure coverage of all relevant 2024 studies. We limited our search to WoSCC for its robust and consistent indexing; while other databases (e.g., Scopus or PubMed) could complement the dataset, using a single comprehensive source ensured uniformity in citation analysis. The search strategy was meticulously designed to ensure specificity and inclusivity, incorporating expert input from clinicians and bibliometricians. The following search terms and Boolean operators were applied: (TS = (“Periprosthetic Joint Infection” OR “PJI” OR “Prosthetic Joint Infection” OR “Infection after joint replacement”) AND TS = (“Biomarker” OR “Biomarkers” OR “Diagnostic Biomarker*” OR “Infection Biomarker*” OR “Molecular Biomarker*” OR “Proteomic Biomarker*” OR “Genomic Biomarker*”). Search filters were employed to limit results to original research articles and reviews written in English, excluding document types such as conference abstracts, editorials, and book chapters. The retrieved records from WoSCC were exported with “Full Record and Cited References”, saved in “plain text” format for subsequent analysis. The initial search identified 394 records; after removing duplicates and non-relevant records, 380 publications remained and were included in the analysis.

### Data extraction

Records retrieved from the WoSCC database included the journals, publication dates, article titles, authors, affiliations, originating countries or regions, abstracts, keywords, publication sources, funding agencies, H-index, and citation frequencies. All records were exported in plain text format and subsequently imported into Microsoft Excel 2021, CiteSpace 6.4.R1, VOSviewer 1.6.20, and R for bibliometric analysis and data visualization.

The H-index, defined as the number of papers (*h*) that have been cited at least *h* times each, was used to evaluate scholarly influence. Journal impact was assessed using the 2023 Journal Citation Reports, noting each journal’s impact factor (IF) and quartile rank (Q1–Q4 in its category). These metrics allowed for an evaluation of journal quality and standing within their respective fields. To ensure data accuracy, two independent researchers screened and extracted key information from the included articles. Any discrepancies in data extraction or classification were resolved through group discussion and consensus. This rigorous approach ensured the reliability and consistency of the data analysis.

### Bibliometric and visualized analysis

We first obtained basic publication metrics directly from WoSCC, including the annual number of publications, total citations per year, and distribution of publication types and languages. For advanced analysis, we constructed bibliometric networks to explore collaborations and thematic relationships. Co-authorship, co-citation, and keyword co-occurrence networks were generated and visualized using VOSviewer and CiteSpace software [13, 14]. These tools helped identify key collaborations, co-citation relationships, and thematic clusters. Complementary analyses were performed using the Bibliometrix and Biblioshiny packages in R, giving further insight and refinement of the core data. These integrated methodologies comprehensively analyze the global research landscape for PJI biomarkers, displaying key trends, collaborations, and hotspots that are gaining momentum.

## Results

### An overview of the annual growth trend

**Fig. 1:**
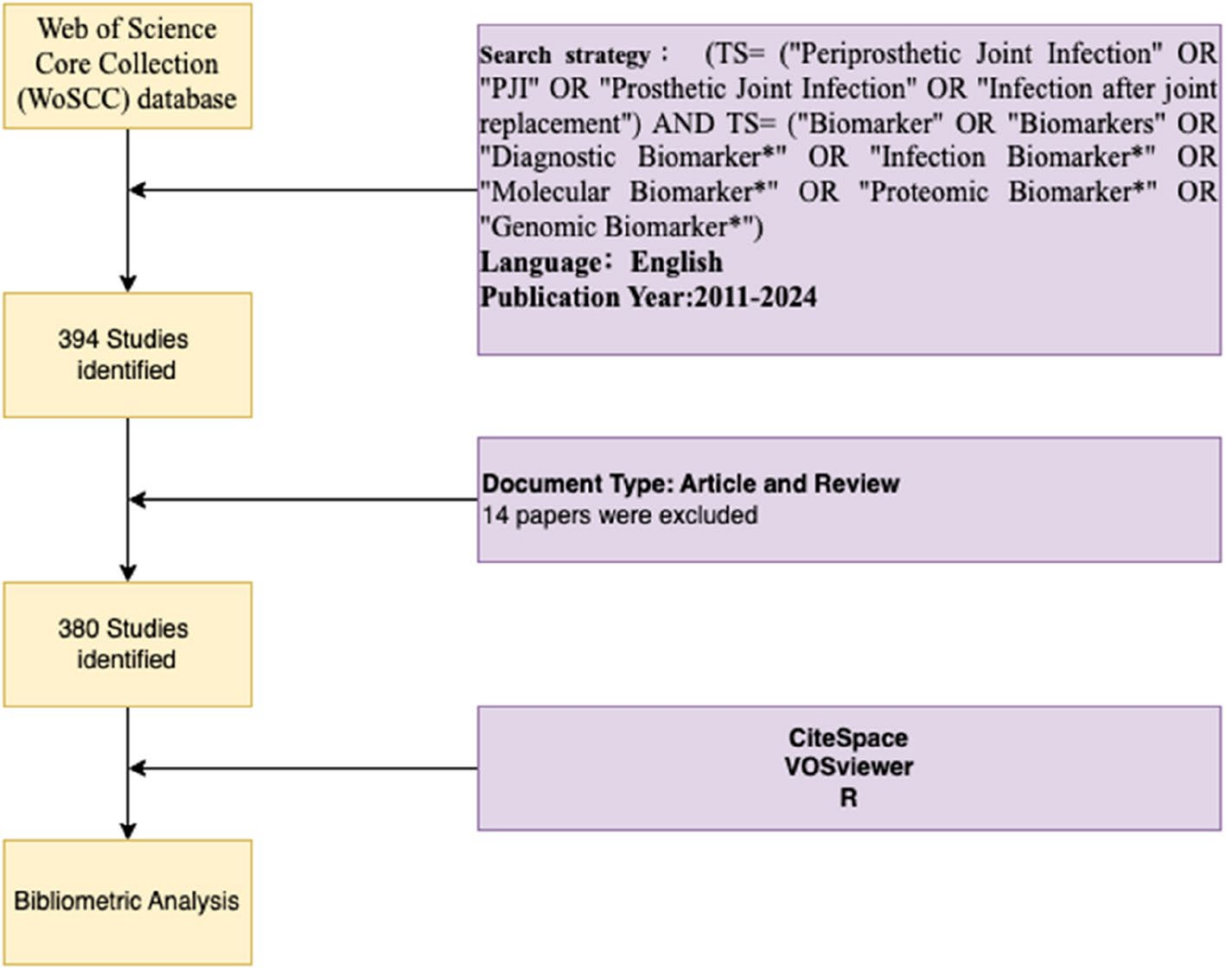
Flowchart of the data screening process

**Fig. 2:**
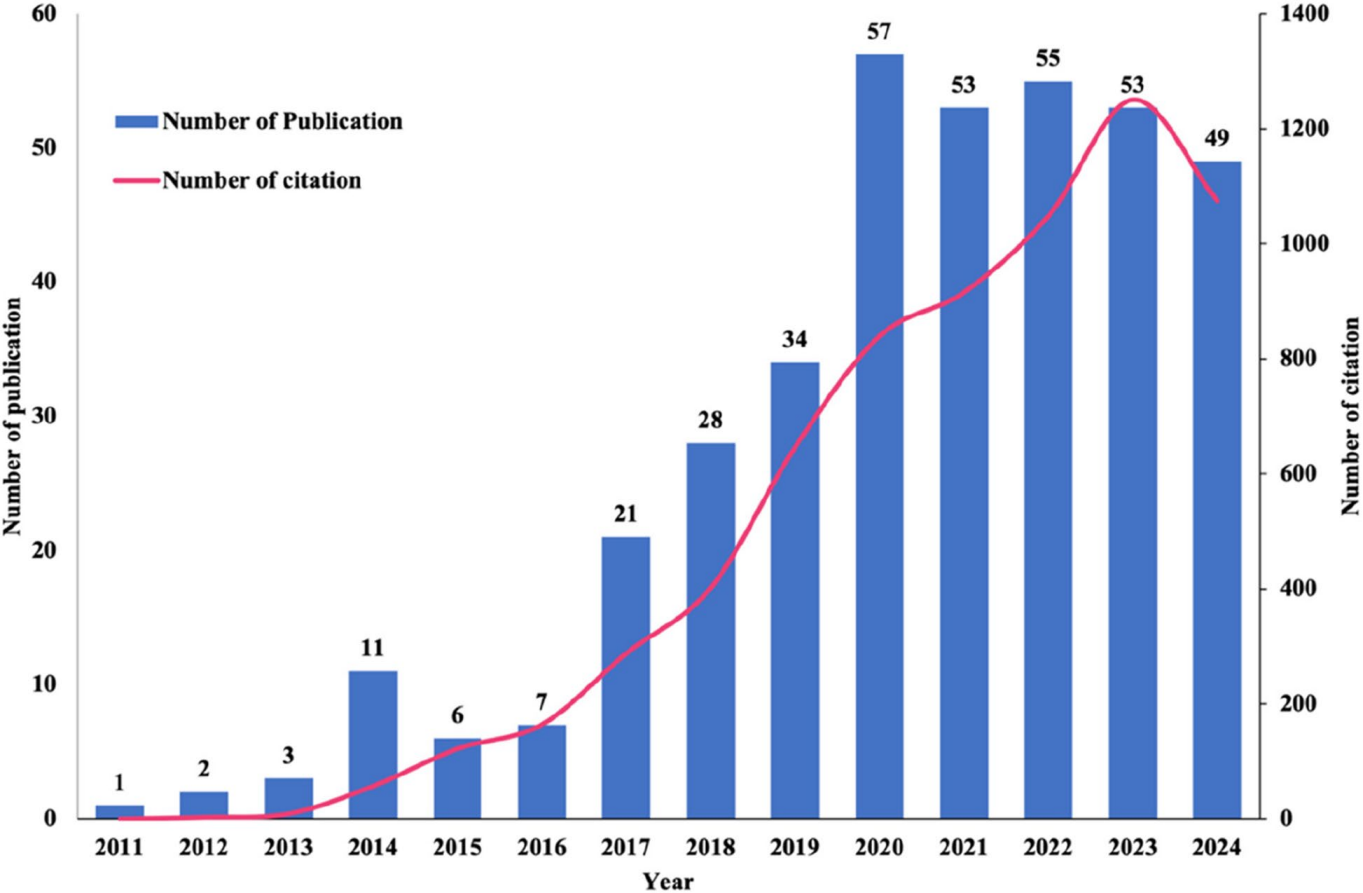
Global trend of annual publications and citations from 2011 to 2024. The bars represent the frequency of publications in each year (x-axis). The line graph shows the total annual citations

A total of 380 eligible publications from the WoSCC database were included in this study until December 31, 2024, including 309 ARTICLES (81%), 71 REVIEWS (19%), as shown in Fig. 1. The annual publication output in this field has increased dramatically over the past decade (2011–2022), reflecting growing academic interest and investment (see Fig. 2 for publication and citation trends by year). From 2011 to 2014, the publications were limited, with fewer than five articles per year. A growth became noticeable in 2015, further accelerated after 2017, peaking at 57 publications in 2020 (an approximate 38% compound annual growth rate since 2011). Citation trends followed the same pattern, with a sharp rise culminating in over 1,200 citations in 2022, showing the growing influence of those studies. Although there is a slight decline in 2023 and 2024, the consistent uptick over the past ten years underlines the ever-expanding relevance of this field and sustained scholarly interest.

### Geographic distribution of publications

**Fig. 3:**
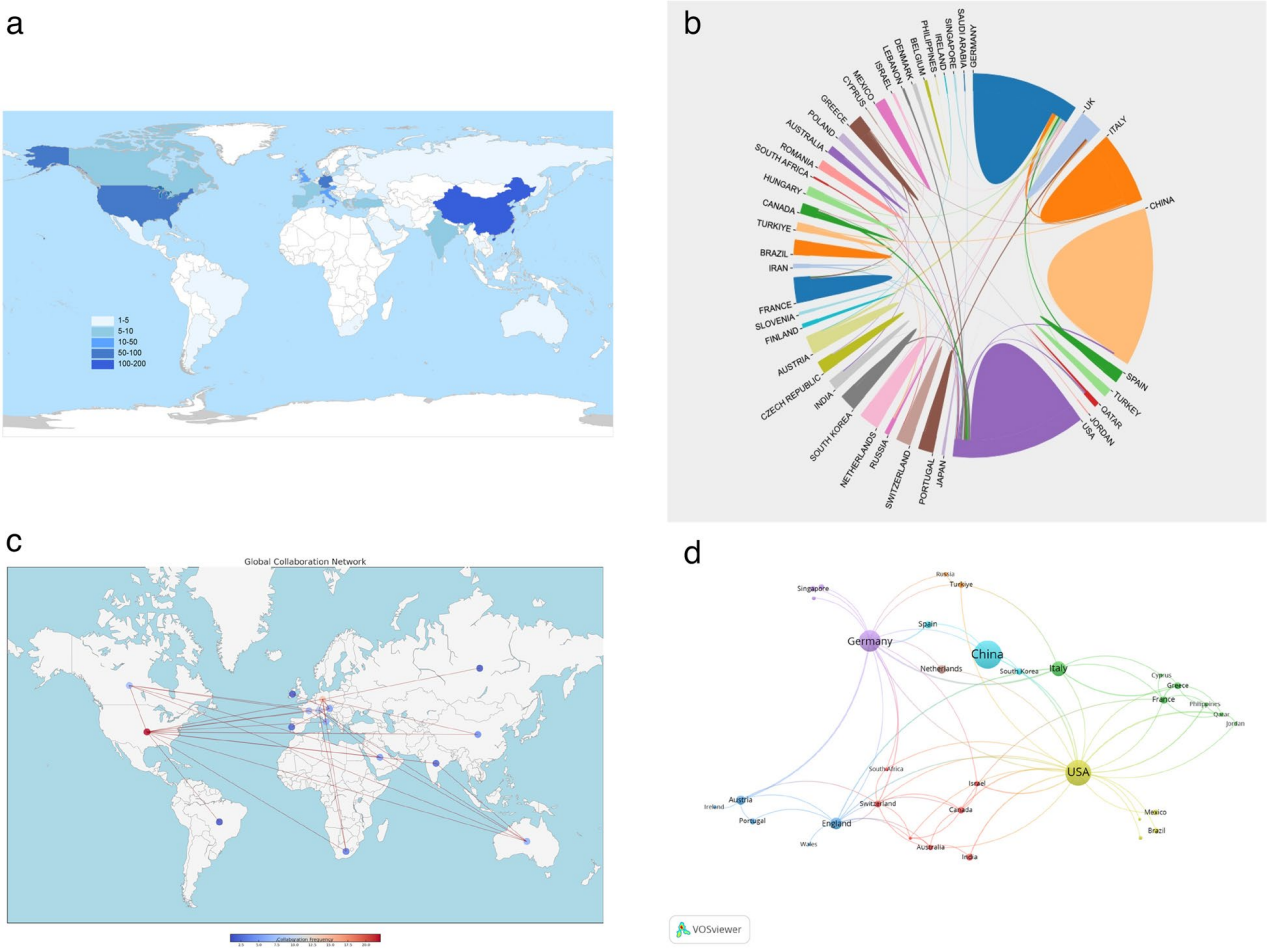
**a** World map of the global collaborations among countries in the field of biomarkers on PJI. **b** Collaborative countries: lists the leading countries with significant cooperative interactions in the research area; (**c**) Country collaboration relationships: illustrates specific collaborative relationships between countries within the research field; (**d**) Collaboration networks among countries or regions based on the VOS viewer. The size of the nodes represents the number of publications from each country/region

As shown in Fig. 3a, the world map displays total outputs by country/region (color-coded by thresholds), with research activity concentrated in North America, Europe, and East Asia. The top 10 contributors (Table 1; Fig. 3a) illustrate a globally distributed effort with notable differences in productivity and impact: China leads with 103 publications (27.11%; H-index 21), followed by the United States with 88 publications (23.16%) and the highest overall influence (2,877 citations, ~33 per paper; H-index 27); China and the USA together contributed over 50% of all publications, reflecting their dominant role in this field. Germany ranks third (60 publications; 15.8%; 951 citations), and Italy and UK complete the top five, with UK showing a strong citation-per-publication ratio (~21). These countries also rank among the annual productivity leaders, while Austria, the Netherlands, and Switzerland achieve strong impact despite smaller volumes. Collaboration mapping (Fig. 3b–d) reveals a dense international network in which the USA serves as a central hub, partnering broadly with European countries and China. China and Germany also maintain substantial bilateral and regional ties. In the visualizations, larger nodes denote dominant contributors and thicker edges indicate stronger co-authorship links. Overall, the patterns highlight a collaborative, global enterprise led by established centers yet increasingly diversified; however, limited participation from many low- and middle-income countries underscores a persistent gap in research inclusivity.

**Table 1:** The top 10 countries/regions with the highest productivity in publications on biomarker research in PJI

Rank	Country	No. of articles	Total citation	% of (380)	Citation per publication	H-index
1	China	103	1372	27.11%	13.32	21
2	USA	88	2877	23.16%	32.69	27
3	Germany	60	951	15.79%	15.85	15
4	Italy	30	329	7.89%	10.97	11
5	UK	18	377	4.74%	20.94	8
6	Austria	12	232	3.16%	19.33	7
7	Netherlands	10	185	2.63%	18.50	7
8	Spain	8	132	2.11%	16.50	5
9	France	8	102	2.11%	12.75	5
10	Switzerland	7	127	1.84%	18.14	5

### Institutional contributions

**Fig. 4:**
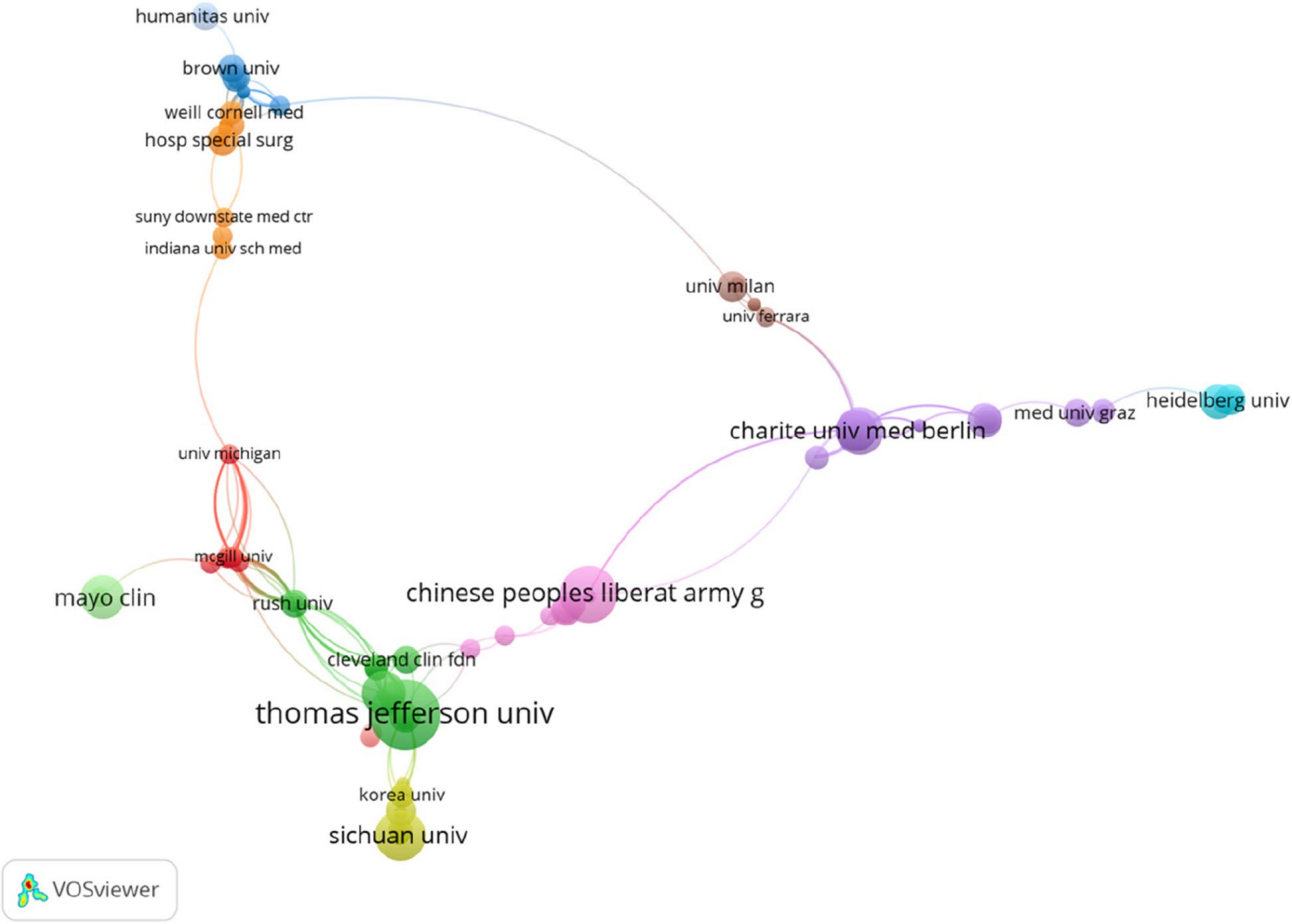
A visual map of institutional collaboration analysis. The size of the nodes represents the number of publications from each institution, and the lines between nodes indicate the strength of collaboration between them

The leading institutions in PJI biomarker research reflect the contributions of major orthopaedic centers predominantly in the *USA*, *China*, and *Europe.* Table 2 lists the top 10 most productive institutions, which together accounted for a substantial portion of the literature. The Rothman Institute (Philadelphia, USA) ranked first with 32 publications (8.4% of all papers). It distinguished itself not only by volume but also by impact, with a total of 1,636 citations and an impressive average of 51 citations per publication. Rothman Institute’s leadership aligns with its pioneering work on synovial biomarker development and its central role in shaping contemporary diagnostic criteria for PJI. Several leading Chinese institutions also appear in the top 10, including the Chinese PLA General Hospital, Sichuan University, and Chongqing Medical University, collectively indicating China’s rising prominence in this domain. In Europe, institutions like Charité – Universitätsmedizin Berlin and the University of Bonn in Germany contributed significantly, each with influential publications (e.g., citation averages of 15–21 per paper). The Mayo Clinic (USA) ranked sixth in output but had one of the highest citation averages (38 per paper), signifying high-impact work despite a moderate number of publications. Other notable contributors included Harvard University, Chang Gung Memorial Hospital, and the Technical University of Munich, each with consistent output. Figure 4 presents the institutional collaboration network visualized using VOSviewer, comprising 43 nodes, each representing a distinct institution. These institutions are grouped into nine color-coded clusters, illustrating collaborative relationships within the field. The connecting lines between nodes signify the strength of cooperation, with denser and thicker lines indicating more active and closer institutional partnerships.

**Table 2:** Top 10 organizations that contributed to publications on biomarker research in PJI

Rank	Organizations	Counts	% of (380)	Total citations	Citations per publication	H-index
1	Rothman Institute	32	8.42%	1636	51.12	17
2	Chinese People’s Liberation Army General Hospital	17	4.47%	178	10.47	8
3	Charité Universitätsmedizin Berlin	14	3.68%	204	14.57	5
4	Sichuan University	13	3.42%	86	6.61	6
5	Chongqing Medical University	11	2.89%	150	13.63	6
6	Mayo Clinic	11	2.89%	418	38.00	9
7	University Of Bonn	9	2.37%	193	21.44	8
8	Chang Gung Memorial Hospital	8	2.11%	75	9.37	5
9	Technical University of Munich	8	2.11%	181	22.62	4
10	Harvard University	7	1.84%	145	20.71	5

### Analysis of journals and co-cited journals

Research on PJI biomarkers spans many journals, but a core group produces nearly half of the literature. The top 10 journals (listed in Table 3) published 170 papers (44.7%). *The Journal of Arthroplasty (JOA)* leads with 48 publications (12.63%) and 1,078 citations (22.46 per article). The *Journal of Orthopaedic Surgery and Research (JOSR)* follows with 21 papers (5.53%) and 316 citations. *The Bone & Joint Journal (BJJ)* ranks third by volume (19) but shows a high citation rate (26.95 per article). *The Journal of Bone and Joint Surgery American Volume (JBJS-Am)* and Clinical *Orthopaedics and Related Research (CORR)* have the highest citation-per-publication (68.5), indicating strong impact. *International Orthopaedics*, *BMC Musculoskeletal Disorders*, and *Bone & Joint Research* contribute steadily, while *Scientific Reports* and *Antibiotics (Basel)* reflect the field’s interdisciplinary reach. Although most listed journals have IFs below 5, they remain established, authoritative outlets in orthopaedics.

**Table 3:** The top 10 productive journals publishing articles on biomarker research in PJI

Ranking	Journal title	Count	Percentage (N/380)	Total citation	Citation per publication	IF (2023)	Quartile in category (2023)
1	Journal of Arthroplasty	48	12.63%	1078	22.46	3.4	Q1
2	Journal of Orthopaedic Surgery and Research	21	5.53%	316	15.05	2.8	Q1
3	Bone Joint Journal	19	5.00%	512	26.95	4.9	Q1
4	International Orthopaedics	17	4.47%	373	21.94	2.0	Q1
5	Journal Of Bone and Joint Surgery American Volume	14	3.68%	959	68.50	4.3	Q1
6	BMC Musculoskeletal Disorders	13	3.42%	103	7.92	2.2	Q2
7	Clinical Orthopaedics and Related Research	12	3.16%	822	68.50	4.2	Q1
8	Bone Joint Research	9	2.37%	214	23.78	4.7	Q1
9	Scientific Reports	9	2.37%	173	19.22	3.8	Q1
10	Antibiotics Basel	8	2.11%	51	6.38	4.3	Q1

**Fig. 5:**
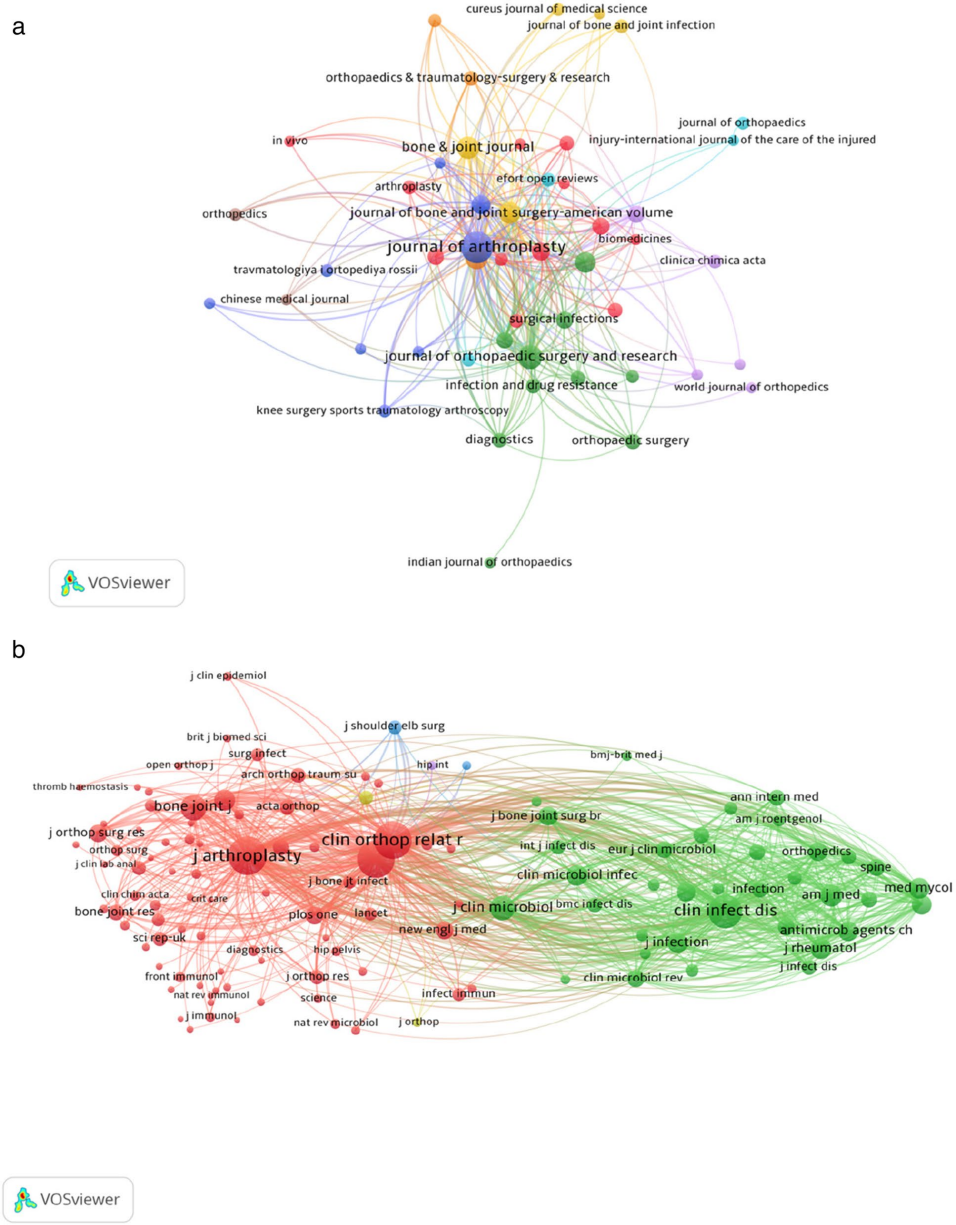
(**a**) The network visualization of journal citation relationships. The size of the nodes indicates the frequency of citations, and the lines between the two nodes represent citations made by a single journal; (**b**) Network visualization of journals’ co-citation relationship

**Fig. 6:**
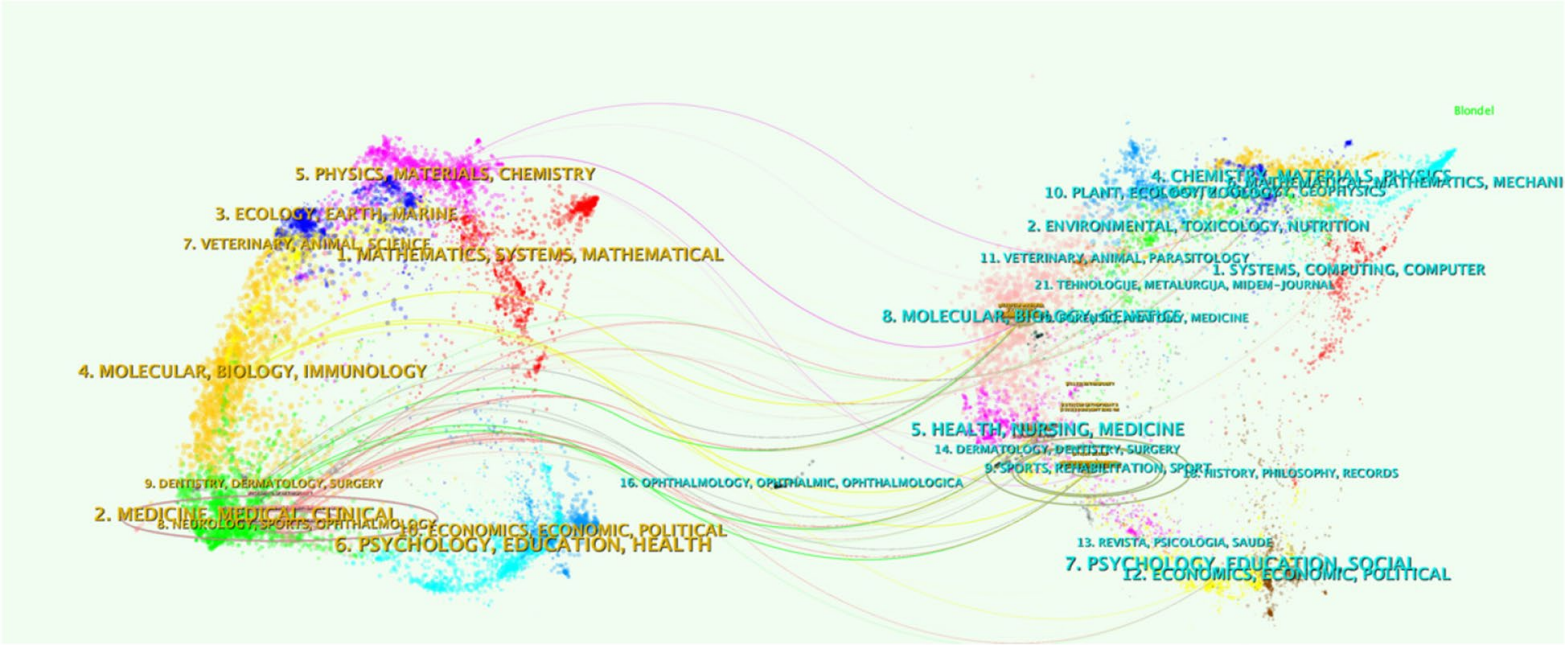
Double journal overlay map of PJI biomarkers. Journals on the right are the cited ones, those on the left are the citing journals, and the colored paths in the center represent the co-citation relationships

The dominance of Q1-ranked journals, such as *JOA, BJJ, JBJS-Am,* and *CORR*, underscores the high-quality research in this field and its publication in well-recognized outlets. The citation and co-citation relationships among these journals were visualized using VOSviewer, as illustrated in Fig. 5a–b. *JOA*, *BJJ*, *JBJS-Am,* and *International Orthopaedics* emerged as central nodes in the network, indicating their substantial influence and close scholarly connections. These journals form the core of knowledge exchange in the field, reflecting their critical role in shaping research and fostering collaboration. A dual-map overlay visualization of journals is displayed in Fig. 6 to further reveal the academic interaction and knowledge dissemination patterns among different journals. The left and right parts represent the cited journals and the citing journals, respectively, which are connected by different colored paths. It can be seen that molecular/biology/immunology, medicine/clinical/medicine, to molecular/biology/genetics, and health/nursing/medicine.

### Authorship and collaboration network

The top 15 most productive authors in the field of PJI biomarker research are listed in Table 4, spanning contributions from the *USA*, *Germany*, and *China*. *Javad Parvizi* (USA) is the most prolific author (26 papers, 1,438 total citations), while *Christopher Deirmengian* (USA) achieved the highest average impact with 96.6 citations per publication (8 papers). Other influential contributors include *Andrej Trampuz* (13 papers, Germany) and leading Chinese researchers such as *Chen Jiying* (12 papers). This reflects a blend of high-output authors and those whose fewer publications have been highly influential across different regions.

**Table 4:** The top 15 productive authors in the field of biomarker research in PJI

Ranking	Author	Country	No. of articles	% of (380)	Total citation	Citation per publication
1	Parvizi J	USA	26	6.84%	1438	55.31
2	Trampuz A	Germany	13	3.42%	200	15.38
3	Chen JY	China	12	3.16%	121	10.08
4	Huang W	China	11	2.89%	148	13.45
5	Hu N	China	10	2.63%	136	13.60
6	Xu C	China	10	2.63%	92	9.20
7	Xu H	China	10	2.63%	57	5.70
8	Chai W	China	9	2.37%	93	10.33
9	Fu J	China	9	2.37%	71	7.89
10	Li R	China	9	2.37%	154	17.11
11	Deirmengian C	USA	8	2.11%	773	96.63
12	Gravius S	Germany	8	2.11%	187	23.38
13	Qin LL	China	8	2.11%	108	13.50
14	Randau TM	Germany	8	2.11%	187	23.38
15	Patel R	USA	7	1.84%	209	29.86

**Fig. 7:**
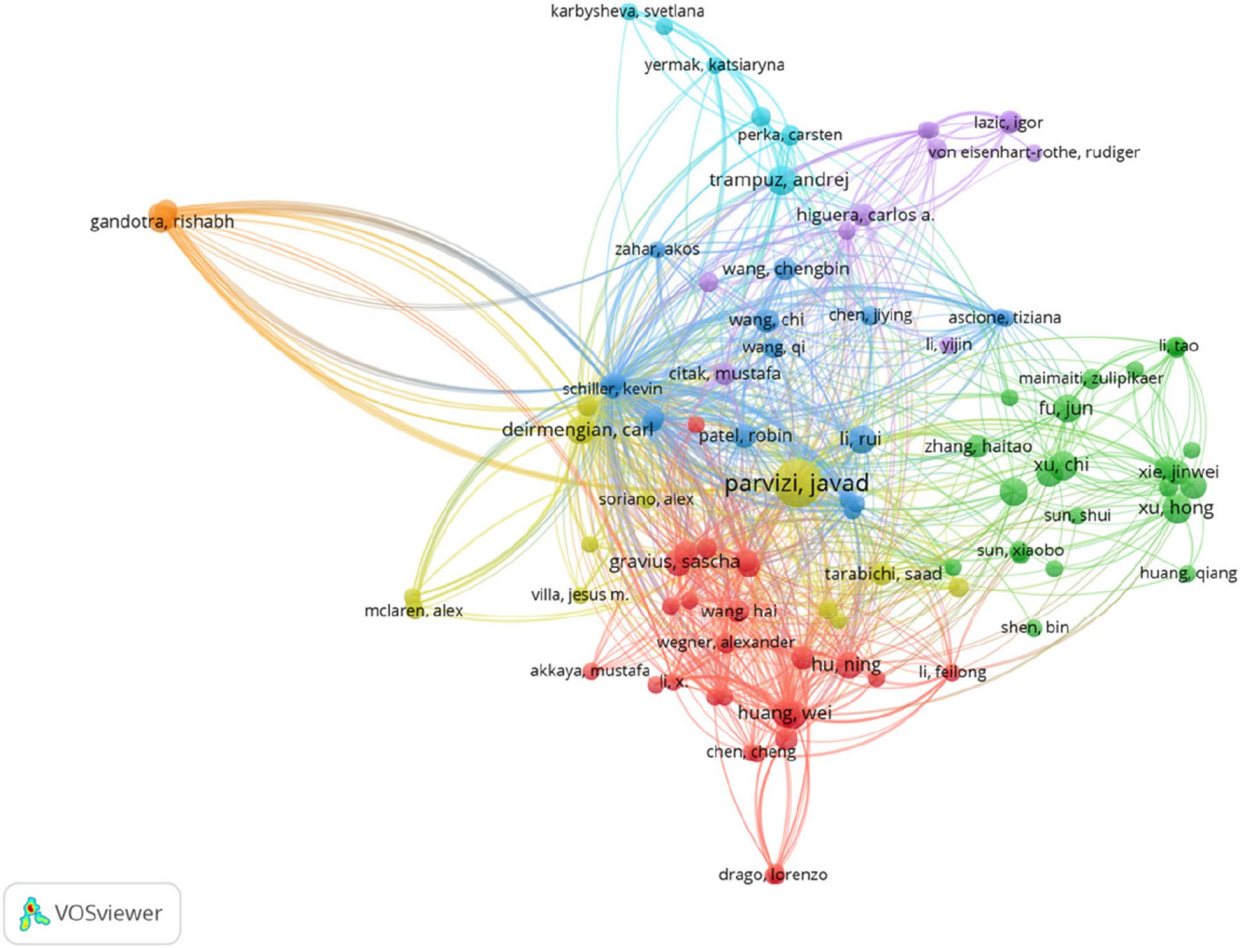
The network visualization map of co-cited authors on biomarker research related to PJI. The size of each node reflects its citation frequency, with each color representing a distinct collaborative research focus group

The co-citation network of authors, visualized using VOSviewer in Fig. 7, reveals clusters of closely related co-citations, with each color representing a distinct group of collaborative research focus. The node size reflects the number of co-citations, highlighting the influence and recognition of an author’s contributions within the field. *Javad Parvizi*, *Andrej Trampuz*, *Chen Jiying*, *Sascha Gravius*, and *Mustafa Citak*, among others, emerge as the most frequently co-cited authors, occupying central positions in the network. At the same time, it highlights international collaboration—many of the clusters include authors from multiple countries, showing active cross-border research partnerships. This collaborative structure bodes well for multi-center studies and consensus-building in the quest for better PJI diagnostics.

### Analysis of cited references

The analysis of the most co-cited articles in PJI biomarker research highlights the significant contributions of key studies in advancing diagnostic methodologies and therapeutic approaches (Table 5). The top three most co-cited articles in PJI biomarker research were authored by *Deirmengian C*, reflecting his substantial influence and foundational contributions to this field. Collectively, these studies, published between 2014 and 2015, have established α-defensin as a cornerstone biomarker for PJI diagnostics, receiving a total of 767 citations. The most highly co-cited article, “Diagnosing Periprosthetic Joint Infection: Has the Era of the Biomarker Arrived?”, published in *CORR* in 2014, received 293 citations. This seminal work introduced the potential of biomarkers, particularly human α-defensin 1–3, neutrophil elastase 2, bactericidal/permeability-increasing protein, neutrophil gelatinase-associated lipocalin, and lactoferrin, as a transformative tool for diagnosing PJI [15]. It highlighted that synovial fluid biomarkers have exhibited high accuracy in diagnosing PJI even in patients with systemic inflammatory diseases or receiving antibiotic therapy. The second-ranked article, “Combined Measurement of Synovial Fluid α-Defensin and C-Reactive Protein Levels: Highly Accurate for Diagnosing Periprosthetic Joint Infection”, published in the *JBJS-Am* in 2014, garnered 245 citations [16]. This study expanded on the utility of α-defensin by combining its measurement with synovial fluid C-reactive protein (CRP) levels. The findings demonstrated high diagnostic sensitivity and specificity, providing a robust, dual-biomarker approach that significantly improved diagnostic accuracy for PJI. The third article, “The Alpha-defensin Test for Periprosthetic Joint Infection Outperforms the Leukocyte Esterase Test Strip”, published in *CORR* in 2015, received 229 citations [17]. This work directly compared the α-defensin test with the leukocyte esterase test strip, a commonly used diagnostic tool, and demonstrated the superior performance of α-defensin in terms of accuracy and reliability. These three studies collectively underscore the pioneering contributions of *Deirmengian* and *Javad Parvizi’s* team in advancing biomarker research for PJI. Their work has set a new standard for precision in PJI diagnostics, particularly in challenging clinical scenarios. By advancing biomarker-based approaches, these studies have addressed critical gaps in PJI detection.

**Table 5:** Top 15 co-cited articles and reviews related to the biomarker research in PJI

Ranking	Title	Article type	Total citations	Frist author	year	journal
1	Diagnosing Periprosthetic Joint Infection: Has the Era of the Biomarker Arrived?	Article	293	Deirmengian, C	2014	Clinical Orthopaedics and Related Research
2	Combined Measurement of Synovial Fluid α-Defensin and C-Reactive Protein Levels: Highly Accurate for Diagnosing Periprosthetic Joint Infection	Article	245	Deirmengian, C	2014	Journal of Bone and Joint Surgery-American Volume
3	The Alpha-defensin Test for Periprosthetic Joint Infection Outperforms the Leukocyte Esterase Test Strip	Article	229	Deirmengian, C	2015	Clinical Orthopaedics and Related Research
4	Synovial Fluid Biomarkers for the Diagnosis of Periprosthetic Joint Infection	Article	164	Lee, YS	2017	Journal of Bone and Joint Surgery-American Volume
5	The Alpha Defensin-1 Biomarker Assay can be Used to Evaluate the Potentially Infected Total Joint Arthroplasty	Article	154	Bingham, J	2014	Clinical Orthopaedics and Related Research
6	The Alpha-Defensin Immunoassay and Leukocyte Esterase Colorimetric Strip Test for the Diagnosis of Periprosthetic Infection: A Systematic Review and Meta-Analysis	Article	145	Wyatt, MC	2016	Journal of Bone and Joint Surgery-American Volume
7	CURRENT CONCEPTS REVIEW Culture-Negative Periprosthetic Joint Infection	Review	152	Parvizi, J	2014	Journal of Bone and Joint Surgery-American Volume
8	Phage Therapy for Limb-threatening Prosthetic Knee Klebsiella pneumoniae Infection: Case Report and In Vitro Characterization of Anti-biofilm Activity	Article	132	Cano, EJ	2021	Clinical Infectious Diseases
9	α-Defensin Accuracy to Diagnose Periprosthetic Joint Infection-Best Available Test?	Article	124	Frangiamore, SJ	2016	Journal Of Arthroplasty
10	Antimicrobial Peptides and Proinflammatory Cytokines in Periprosthetic Joint Infection	Article	128	Gollwitzer, H;	2013	Journal of Bone and Joint Surgery-American Volume
11	Can next-generation sequencing play a role in detecting pathogens in synovial fluid?	Review	112	Tarabichi, M	2018	Bone & Joint Journal
12	Interleukin-6 in Serum and in Synovial Fluid Enhances the Differentiation between Periprosthetic Joint Infection and Aseptic Loosening	Article	112	Randau, TM	2014	Plos One
13	Serum biomarkers in periprosthetic joint infections	Article	98	Saleh, A	2018	Bone & Joint Research
14	Diagnosis of Periprosthetic Joint Infection Using Synovial C-Reactive Protein	Article	88	Parvizi, J	2012	Journal of Arthroplasty
15	Twenty common errors in the diagnosis and treatment of periprosthetic joint infection	Review	88	Li, C	2020	International Orthopaedics

The fourth-ranked co-cited article is Lee et al. (2017) in *JBJS-Am*, *“Synovial Fluid Biomarkers for the Diagnosis of PJI,”* which garnered 164 citations. Lee and colleagues provided a comparative evaluation of various synovial biomarkers (including α-defensin) in diagnosing PJI, offering valuable guidance on selecting optimal synovial tests in clinical practice [18]. The fifth is *Bingham* et al. (2014) in *CORR*, which validated the α-defensin-1 immunoassay in a clinical setting, demonstrating its reliability for evaluating suspected infected joint arthroplasties [19]. This study (154 citations) further reinforced the diagnostic utility of α-defensin-1 and contributed to the evidence base that supported its adoption in practice.

The remaining 10 articles in the co-citation analysis reflect the diverse advancements and ongoing efforts in PJI biomarker research, focusing on both diagnostic methodologies and innovative approaches to managing infections. *Wyatt* et al. (2016) performed a meta-analysis of 11 studies and showed that the synovial α-defensin immunoassay achieved a pooled sensitivity of 100% and specificity of 96%, while the leukocyte esterase strip test showed 81% sensitivity and 97% specificity, confirming both as highly accurate PJI biomarkers, with α-defensin marginally superior [20]. *Parvizi* et al. reviewed culture-negative PJI, identifying pre-aspiration antibiotics, suboptimal sample transport, implant sonication, prolonged incubation, and recommending integration of molecular techniques (PCR/NGS) with synovial biomarkers to improve detection [21]. *Cano* et al. reported a refractory Klebsiella pneumoniae prosthetic knee infection successfully treated with 40 intravenous doses of phage KpJH46Φ2 plus minocycline, resulting in symptom resolution, sustained asymptomatic status at 34 weeks, and an in vitro biofilm reduction trend (*P* = 0.063) [22]. *Frangiamore* et al. compared laboratory and point-of-care α-defensin assays in revision arthroplasty, finding both formats > 90% accurate but differing in turnaround time, and confirmed α-defensin’s superior sensitivity and specificity over conventional tests [23]. *Gollwitzer* et al. measured synovial LL-37, HBD-3, and cytokines (IL-1β, IL-4, IL-6, IL-17A, IFN-γ, TNF-α) in 15 PJI versus 20 aseptic cases, reporting AUCs up to 0.972 for combined HBD-3 + IL-4 and demonstrating that synovial peptides and cytokines outperform serum markers [24]. *Tarabichi* et al. evaluated NGS in 86 synovial fluid samples, showing concordance with culture in 25/30 positive cases, detection of antibiotic-resistant organisms missed by culture, and identification of pathogens in 10 culture-negative samples, concluding NGS is a valuable adjunct [25]. *Randau* et al. assessed IL-6 in serum and synovial fluid in a large prospective cohort, finding synovial IL-6 > 2100 pg/mL yielded 85.7% specificity and 59.4% sensitivity (rising to nearly 100% specificity at > 9000 pg/mL), and serum IL-6 > 6.6 pg/mL achieved 88.3% specificity, supporting synovial IL-6 as more accurate than serum CRP and WBC [26]. *Saleh* et al. reviewed serum biomarkers and confirmed that ESR and CRP remain first-line tests, that D-dimer shows promise, but no new serum markers yet surpass conventional assays, noting factors (timing, metallosis) that influence levels [27]. *Parvizi* et al. prospectively measured synovial CRP in 63 arthroplasty patients, finding a mean of 40 mg/L in septic versus 2 mg/L in aseptic cases (*P* < 0.0001), with 85% sensitivity and 95% specificity at a 9.5 mg/L cutoff (AUC 0.92), endorsing synovial CRP as a reliable marker [28]. *Li* et al. systematically outlined twenty frequent diagnostic and management errors—including misuse of serum biomarkers to exclude PJI, inadequate sampling, suboptimal microbiology, and failure to individualize treatment—and advocated multidisciplinary collaboration to avoid these pitfalls [29].

**Fig. 8:**
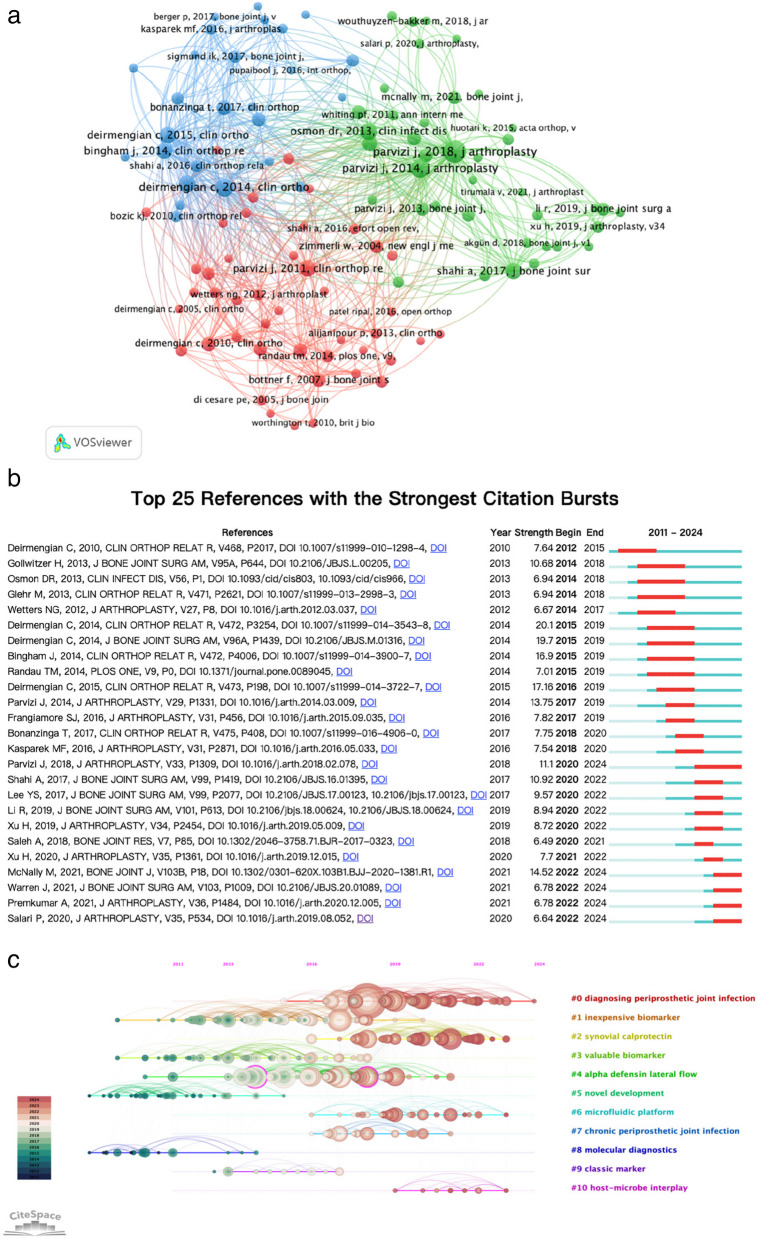
(**a**) Network map of co-cited references. The size of each node indicates the frequency of citations, and the lines connecting two nodes represent references that are cited together in the same paper; (**b**) Cluster view, top 25 references with strongest citation bursts based on Cite Space; (**c**) Timeline visualization from 2011 to 2024, the position of the nodes on the horizontal axis indicates the time when the reference first appeared, the 10 clusters are marked and color coded on the right side, the nodes are large and dense indicating which was the hot topics in that time period

To better capture the intellectual structure of PJI diagnostics research, we extracted 110 co-cited references with at least 20 citations and mapped them using VOSviewer (Fig. 8a). In this network, *Parvizi J* and *Deirmengian C* occupy the most central, highly interconnected positions, each anchoring a distinct cluster of studies. Figure 8b presents each reference’s active citation window (blue bars) alongside periods of unusually strong citation growth (red bursts). The first burst appeared in 2012 for *Deirmengian C* 2010 (*CORR*) [30], while the most intense bursts between 2015 and 2019 include two *Deirmengian* 2014 papers and *Bingham J* 2014 [15, 16, 19]. Notably, the latest bursts extending into 2022–2024 are driven by *McNally M* 2021 (*BJJ*) and *Warren J* 2021 (*JBJS-Am*) [31, 32]. Finally, the CiteSpace timeline view (Fig. 8c) delineates eleven thematic clusters: early hotspots (#1 inexpensive biomarker, #2 synovial calprotectin) peaked around 2012–2014; mid-term themes (#4 alpha defensin lateral flow, #8 molecular diagnostics) dominated 2015–2018; and the most recent fronts (#0 diagnosing periprosthetic joint infection, #5 novel development, #10 host–microbe interplay) have grown steadily from 2019 through 2024.

### Keywords analysis of research hotspots

**Fig. 9:**
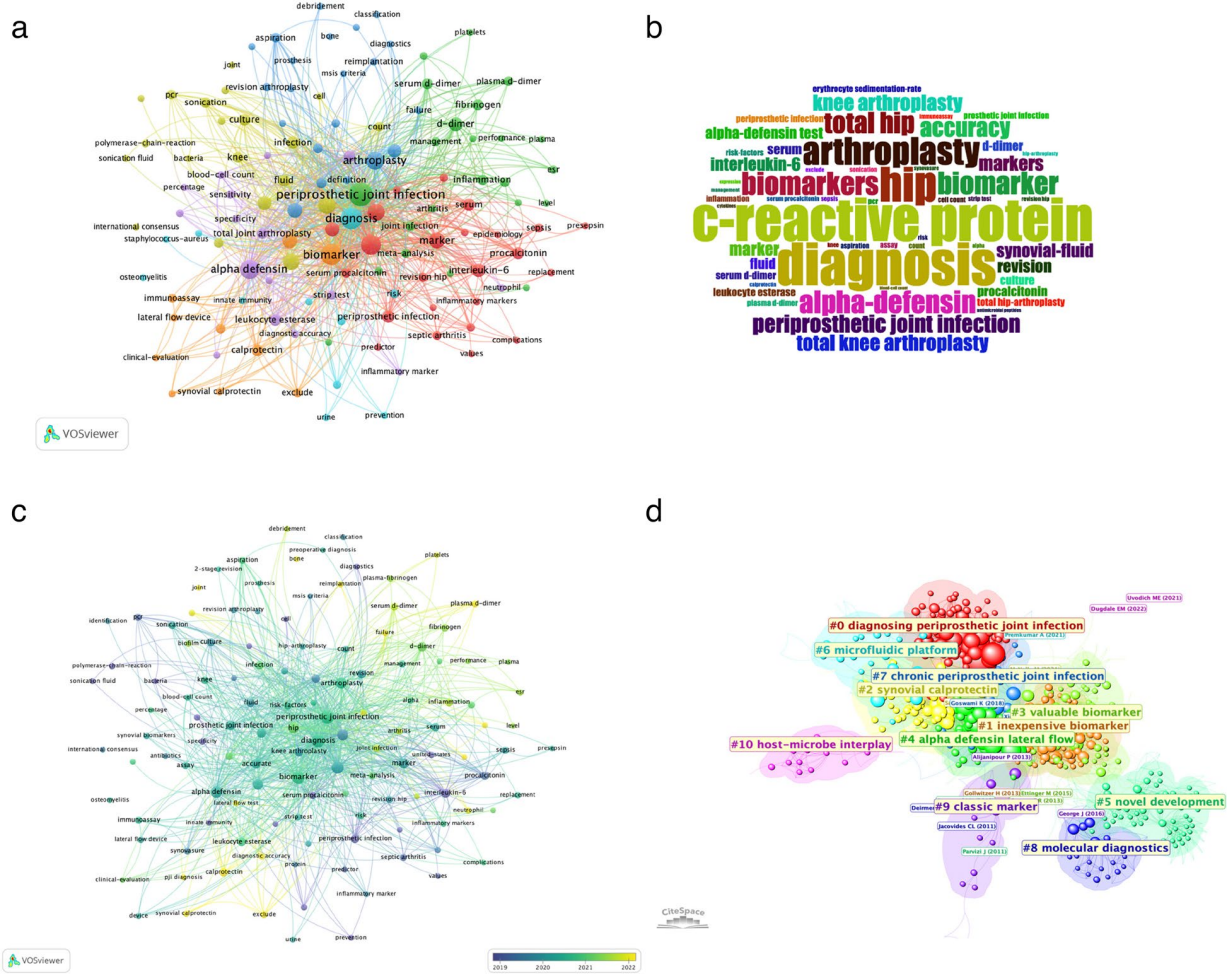
**a** Network visualization of keywords based on the VOS viewer. The size of the nodes indicates the frequency of keyword occurrences, while the lines connecting the nodes represent the co-occurrence frequency between keywords; (**b**) A word cloud of frequently occurring keywords in PJI biomarker publications (2011–2024); (**c**) Overlay visualization of keyword co-occurrence network by average publication year (blue = earlier, yellow = later); (**d**) Network visualization of PJI biomarker keyword clustering. The labels correspond to the names of the clusters formed based on keyword similarities

To enhance readability, the seven keyword clusters (Fig. 9a) were collapsed into three domains. Conventional domain aggregates classic inflammatory and procedural terms, including CRP, ESR, leukocyte esterase, arthroplasty, aspiration, debridement, MSIS/consensus criteria, and routine microbiology (culture, sonication, PCR), capturing established diagnostic practice. The Emerging domain centers on synovial immuno-inflammatory markers and accessible serology, led by α-defensin, calprotectin, synovial IL-6, and blood-based D-dimer/fibrinogen, consistent with the word-cloud prominence of “diagnosis,” “C-reactive protein,” and “α-defensin” (Fig. 9b). The Translational/technology-driven domain comprises mNGS/nanopore, microfluidic platforms, machine learning, and point-of-care testing, alongside host–microbe interplay and guideline terms that frame clinical implementation. The temporal overlay (Fig. 9c) follows a coherent progression from Conventional (2015–2017) to Emerging (2018–2020) and then Translational (2021–2024), while CiteSpace clusters map (Fig. 9d) onto these domains (e.g., #9 “classic marker” and #7 “chronic PJI” to Conventional; #2 “synovial calprotectin,” #4 “α-defensin lateral flow,” #1 “inexpensive biomarker” to Emerging; #6 “microfluidic platform,” #8 “molecular diagnostics,” #5 “novel development” to Translational). Keyword-burst detection (Supplementary Figure S1) aligns with this trajectory, moving from early (pre-2016) innate-immunity signals (procalcitonin, interleukin-6, antimicrobial peptides) to consolidation around α-defensin/Synovasure (2017–2019) and, more recently, coagulation-related serology (fibrinogen, D-dimer) and computation/bedside technologies (machine learning, microfluidic, point-of-care). Collectively, Fig. 9a–d and Supplementary Fig. S1 delineate both the field’s pillars and its forward edge under a unified three-domain framework.

## Discussion

The bibliometric findings illustrate a rapidly expanding research focus on PJI biomarkers over the past decade. Publication output grew dramatically from only a handful of papers in 2011 to a peak of 57 papers in 2020, accompanied by a surge in citations by 2022. This trend reflects the increasing clinical imperative for better diagnostic tools in PJI and coincides with the emergence of novel biomarker assays and updated consensus criteria in 2018. The sustained scholarly interest underscores that PJI diagnosis remains a critical challenge driving global research efforts.

Geographically, research productivity is concentrated in a few leading countries. China and the USA together contributed roughly half of all PJI biomarker publications, with China leading in sheer output (27% of papers) and the USA in cumulative citations and H-index. Germany, UK, and Italy also rank among the top contributors. This dominance by high-resource countries has fostered strong international collaborations, such as the USA serves as a central hub linking European and Asian partners. However, the limited participation from low- and middle-income countries highlights a gap in global representation. Broader inclusion of these regions in future research is essential to ensure that biomarker advances are applicable worldwide and address diverse pathogen profiles, such as mycobacterial or fungal PJIs in certain regions. This disparity likely stems from resource and infrastructure limitations; increasing support for research in LMICs is important to ensure new biomarker techniques are validated across all regions and relevant pathogen profiles. The author and co-citation analyses highlight that a relatively small group of experts and centers has disproportionately shaped the field. *Javad Parvizi* and colleagues stand out for their prolific and impactful work; their research collective has produced seminal findings, from establishing novel biomarkers to improving diagnostic criteria. Similarly, the network of European researchers centered around *Andrej Trampuz* has significantly contributed to both diagnostics and consensus definitions for PJI. The collaboration patterns indicate that these experts often work across international lines, which has facilitated multi-center studies and perhaps quicker validation of new biomarker tests in different settings.

The analysis of journals and citations suggests that PJI biomarker research has achieved mainstream visibility in high-impact orthopedic literature. Nearly 45% of papers appeared in the top 10 journals, led by the *JOA* (12.6% of all publications). Other key outlets include *JOSR*, *BJJ*, *JBJS-Am*, and *CORR*, which notably show very high citation rates per article. The prominence of these Q1 orthopedic journals indicates that research in this field is of high quality and clinical relevance. At the same time, the presence of publications in general science and infectious disease journals (e.g., *Scientific Reports*, *Antibiotics*) highlights the interdisciplinary nature of PJI biomarker studies, bridging orthopedic surgery, microbiology, and immunology.

Over the past decade, the focus of PJI biomarker research has gradually shifted in response to clinical needs and technological developments. Early work concentrated on optimizing conventional inflammatory markers (CRP, ESR) and exploring readily available tests (e.g., leukocyte esterase strips) to compensate for the limitations of culture-based diagnosis. Subsequent studies moved toward synovial biomarkers and molecular techniques, with synovial α-defensin representing a breakthrough by achieving high accuracy, including in some culture-negative cases, and demonstrating that molecular diagnostics can outperform traditional methods [15–17]. Building on this concept, investigators evaluated other synovial markers such as interleukin-6 (IL-6), calprotectin, and antimicrobial peptides including LL-37, HBD-3, NGAL, lactoferrin, and MNDA, as well as cytokine panels that combine multiple inflammatory mediators. Collectively, these markers have shown strong diagnostic performance in research settings; however, none have yet displaced conventional serum CRP/ESR as first-line tests, which underscores the importance of integrating multiple markers into composite algorithms rather than relying on a single “gold standard.”

Our keyword analysis reflects these trends, with terms related to synovial fluid analysis and immunoassays increasingly prominent. More recently, the appearance of “machine learning” and “NGS” indicates a transition toward technology-driven diagnostics. Although host-response biomarkers have improved sensitivity, definitive microbiological identification remains crucial. Traditional culture carries false-negative rates up to 30%, particularly after antibiotic exposure, prompting interest in molecular diagnostics. Early PCR and 16S rRNA sequencing offered modest gains, but metagenomic next-generation sequencing (mNGS) represented a major advance. In 2018, Tarabichi et al. detected pathogens in 10 culture-negative PJI cases—including fastidious or antibiotic-suppressed organisms—using mNGS [25], and a 2024 study reported 89% sensitivity and 95% specificity, identifying unexpected organisms in over 40% of culture-negative cases [33]. Most recent studies indicate that mNGS substantially improves pathogen detection in culture-negative PJI and can identify atypical organisms and resistance determinants, while emerging nanopore workflows further shorten turnaround times toward clinically actionable intraoperative windows [34, 35]. Likewise, integrating emerging tools such as machine learning (ML) algorithms into clinical practice, preliminary ML models using gradient-boosting and neural-network architectures have achieved accuracies comparable to traditional scoring systems in PJI prediction, for example, in primary total knee arthroplasty cohorts [36, 37]. Looking forward, integrated decision-support platforms are being developed to synthesize biomarker panels, imaging findings, histopathology, and genomic data into real-time PJI probability scores, thereby augmenting surgeon judgment in complex revision cases.

Despite its diagnostic potential, mNGS adoption remains constrained by high cost, the need for specialized bioinformatics support, prolonged turnaround times, and the absence of standardized interpretation frameworks. Similarly, machine-learning approaches require rigorous validation, regulatory clearance, and standardized training before they can be reliably integrated into clinical workflows. Clinical applicability ultimately depends not only on diagnostic accuracy but also on cost, availability, and performance in challenging scenarios such as low-grade or culture-negative PJI. These practical limitations—including assay expense, infrastructure demands, variability in thresholds, challenges in reproducibility, and regulatory hurdles—help explain why promising biomarkers have not yet supplanted conventional tests in routine diagnostic pathways.

The trend toward intraoperative diagnostics has spurred the development of point-of-care (POC) assays. Lateral-flow α-defensin and calprotectin tests now provide near-lab-quality results in minutes, enabling surgeons to make informed decisions on single-versus two-stage exchanges [38]. Some studies have reported that synovial calprotectin has progressed to prospective validation and routine POC use, with pooled sensitivity and specificity around 0.9, enabling rapid, same-visit decisions [39, 40]. Future innovations include microfluidic “lab-on-a-chip” devices capable of multiplex analyses from minute fluid volumes—promising comprehensive POC panels that seamlessly integrate into surgical workflows [41]. However, these POC formats, but access may still have limited access, and performance can vary depending on local pre-analytic handling and case-mix. The 2018 ICM/MSIS criteria marked a watershed by formally incorporating biomarkers into a weighted point-based diagnostic algorithm. Tests like α-defensin, synovial CRP, and D-dimer were assigned point values based on evidence strength, alongside traditional major criteria (e.g., sinus tract, positive culture). The 2021 EBJIS definition further refined this approach with a three-tier “traffic light” system—categorizing cases as infection unlikely, likely, or confirmed—to reduce indeterminate diagnoses. Our findings mirror these developments: the prominence of α-defensin (and D-dimer) research aligns with their incorporation into such consensus criteria, whereas cutting-edge tools like microfluidic platforms, mNGS, and ML, not yet included in current guidelines, represent promising avenues for future consensus updates.

Building on the bibliometric hotspots identified in this study, recent breakthroughs and emerging technologies in PJI biomarker research are reshaping current clinical practice in several translational domains: Host-side panels are moving beyond single markers, with synovial IL-6 combinations and metabolomic readouts such as D-lactate improving discrimination in indeterminate cases [42, 43]. Integrated ML models that combine clinical, serologic, and synovial features now provide calibrated case-level probabilities suitable for decision support[44] and platform advances, including microfluidic lab-on-a-chip multiplexing and droplet digital PCR for low-copy targets, support rapid organism and resistance-gene calls[45, 46]. To translate these gains, the field should expand global data-sharing consortia for validation across diverse settings, integrate multimodal algorithms (clinical factors, synovial/serum markers, imaging, genomics) into real-time AI-driven (machine learning) decision support, standardize assay protocols and thresholds within consensus guidelines through multicenter trials and meta-analyses, and rigorously evaluate cost-effectiveness while developing low-cost, point-of-care options to ensure equitable access.

## Limitations

This study has several limitations. First, restricting the search to WoSCC and English-language publications may have led to the omission of studies indexed in other databases or regional, non-English work, although the main trends are unlikely to change. This may introduce some bias, and citation counts themselves can be skewed by self-citation or regional citation practices. Second, citation-based metrics favor older publications (and the slight dip in 2023–2024 output is likely due to indexing delays) and may not reflect clinical impact; because older articles have had more time to accumulate citations, recently published but potentially influential work may be systematically underrepresented. Our burst/recency analyses mitigate this, but very new developments may still be undercaptured. Third, co-occurrence and clustering entail subjective choices; triangulation across tools and manual checks reduces, but does not eliminate, overlap and ambiguity. Fourth, bibliometric signals do not necessarily translate to improved patient outcomes; real-world impact depends on assay availability and affordability, regulatory and laboratory standardization, workflow integration, and external multicenter validation with prospective and cost-effectiveness evidence. To frame recent advances, the Discussion includes brief 2025 citations, without altering the conclusions. Despite these constraints, dual independent extraction and established bibliometric software provide a robust, high-level view of key trends and influential contributions.

## Conclusions

This bibliometric analysis shows a substantial expansion of PJI biomarker research over the past decade, with major contributions from the USA, China, and Europe. Synovial α-defensin marked an important diagnostic advance, followed by calprotectin and other emerging synovial assays. Evolving keyword patterns indicate increasing attention to machine learning, microfluidics, and next-generation sequencing. Going forward, the field should prioritize multicenter validation of promising biomarkers, integration of these markers into evidence-based diagnostic guidelines, and robust cost-effectiveness evaluations. Standardizing assay procedures and cut-off values will also be essential**,** alongside ensuring global accessibility and the development of affordable point-of-care options for low-resource settings. These efforts will help accelerate clinical translation and support earlier, more reliable diagnosis of PJI.

## Supplementary Information

Supplementary Material 1.

## Data Availability

No datasets were generated or analysed during the current study.
