# Supplementary figures and images for: Global trends in biomarker research for periprosthetic joint infection: a bibliometric analysis
Source: Arthroplasty. 2026 Jan 27;8:7. doi: 10.1186/s42836-025-00359-2 (PMC12838467; doi:10.1186/s42836-025-00359-2)

**Supplementary figure 1.**

**
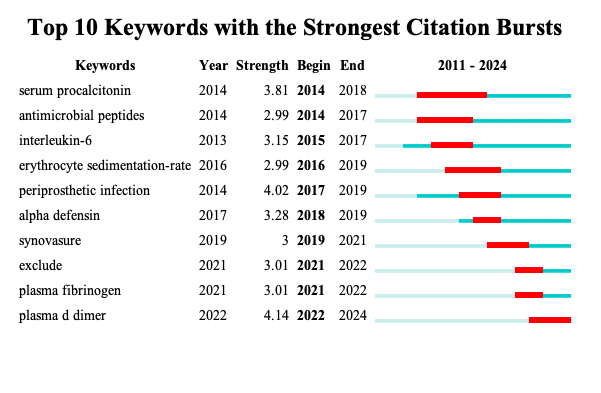
**

Top 10 Keywords with strongest citation burst

Supplement: Supplementary file 2 — Supplementary Material 1. [file 42836_2025_359_MOESM1_ESM.docx]
